# 

**Published:** 1896-06

**Authors:** 


*The following donations have been received since the publication of the list in March:*

*May 30th, 1896.*

| | |
|---|---|
| C. W. J. Brasher (1) | 1 volume. |
| L. M. Griffiths (2) | 4 volumes. |
| Laryngological Society of London (3) | 2 ,, |
| Royal Academy of Medicine in Ireland (4) | 1 volume. |
| Royal College of Surgeons of England (5) | 1 ,, |
| James Swain, M.D. (6) | 1 ,, |
| J. Wright & Co. (7) | 1 ,, |

Unbound periodicals have been received from Dr. James Swain. Framed pictures have been presented by Mr. F. R. Cross, Mr. G. Barry Hart, and Mr. H. E. Hetling.

### TWENTIETH LIST OF BOOKS.

The titles of books mentioned in previous lists are not repeated.

The figures in brackets refer to the figures after the names of the donors, and show by whom the volumes were presented. The books to which no such figures are attached have either been bought from the Library Fund or received through the *Journal.*

**Allingham, W. and H. W.** *Diseases of the Rectum* ... ... ... 6th Ed. 1896
**Ballantyne, J. W.** *Curettage of the Uterus* ... ... ... ... ... ... ... 1896
**Bell, R.** ... ... *Sterility* ... ... ... ... ... ... ... ... ... ... ... 1896
**Binz, C.** ... ... *Lectures on Pharmacology.* Vol. I. (Tr. from 2nd German Ed. by A. C. Latham) ... ... ... 1895
**Boerhaave, [H.]** ... *Aphorisms* (Translated) ... ... ... ... ... (1) 1742
**Boisliniere, L. C.** *Obstetric Accidents, Emergencies, and Operations* ... ... 1896
**Brothers, A.** ... *Infantile Mortality during Child-birth and its Prevention* 1896
**Buret, F.** ... ... *Syphilis in the Middle Ages and in Modern Times* (Tr. by A. H. Ohmann-Dumesnil) ... ... ... ... 1895
**Collins, E. T.** ... *Researches into the Anatomy and Pathology of the Eye* 1896
**Duncan, A.** ... ... *Memorials of the Faculty of Physicians and Surgeons of Glasgow*, 1599-1850 ... ... ... ... ... ... ... 1896
**Eccles, W. McA.** ... *Elementary Anatomy and Surgery for Nurses* ... ... 1896
**Gant, S. G.** ... ... *Diseases of the Rectum, Anus, and Contiguous Textures* 1896
**Gardner, H. B.** ... *The History of Surgical Anæsthesia* ... ... ... ... [1896]
**Gubb, A. S.** ... *Le Placenta dans la Grossesse extra-utérine* ... ... (6) 1893
**Harvey, W.** ... *Prelectiones Anatomiæ Universalis*, [1616] ... ... (5) 1886
**Hedley, W. S.** ... *Current from the Main* ... ... ... ... ... ... ... ... 1896
**Jessop, C. M.** ... *Dress and Health* ... ... ... ... ... ... ... ... ... ... 1896
**Kenwood, H. R.** ... *Public Health Laboratory Work* ... ... ... 2nd Ed. 1896
*Lexicon of Medicine and the Allied Sciences* (New Syd. Soc.). Pin—Puke... 1895
**McFarland, J.** ... *A Text-Book upon the Pathogenic Bacteria* ... ... ... 1896
**Mellin, G.** [Ed.]... *The Feeding of Infants and Growing Children* ... ... 1895
**Miller, A. G.** ... *On the Diagnosis of Tubercular Joint Disease* ... ... 1896
**Moure, E. J.** ... *Du Traitment chirurgical de la Surdité et des Bourdonnements* ... ... ... ... ... ... ... ... ... 1896
**Murray, W.** ... *Rough Notes on Remedies* ... ... ... ... ... ... ... 1896
*Obstetrics, An American Text-Book of* (Ed. by R. C. Norris). 2 vols. ... 1896
*Oeynhausen und seine Indicationen* ... ... ... ... ... ... ... ... ... ... [N.D.]
*Pharmacopœia of the Evelina Hospital for Sick Children*... ... ... 2nd Ed. 1895
**Prichard, A.** ... *A Few Medical and Surgical Reminiscences* ... ... ... 1896
**Quain, [J.]** ... ... *Elements of Anatomy.* 10th Ed. (Ed. by E. A. Schäfer and G. D. Thane). ... ... ... Appendix 1896
**Roberts, C.** ... ... *The Medical Inspection of, and Physical Education in, Schools* ... ... ... ... ... ... ... ... ... 1896
**Sibley, W. K.** ... *State Aided* v. *Voluntary Hospitals* ... ... ... ... 1896

| | | |
|---|---|---|
| **Sutton, J. B.** ... | *Surgical Diseases of the Ovaries and Fallopian Tubes, including Tubal Pregnancy* ... ... ...New Ed. | 1896 |
| **Thompson, W. G.** | *Practical Dietetics* ... ... ... ... ... ... ... ... ... | 1896 |
| **Unna, P. G.** ... | *The Histopathology of the Diseases of the Skin* (Tr. by N. Walker) ... ... ... ... ... ... ... ... | 1896 |
| **[Zaehnsdorf, J.]** ... | *A Short History of Bookbinding* ... ... ... ... ... | 1895 |

---

### TRANSACTIONS, REPORTS, JOURNALS, &c.

| | | |
|---|---|---|
| Alienist and Neurologist, The... ... ... ... ... ... ... ... | Vol. XVI. | 1895 |
| American Dermatological Association, Transactions of the ... ... ... | | 1896 |
| American Gynecological Society, Transactions of the ... ... | Vol. XX. | 1895 |
| American Laryngological Association, Transactions of the ... ... ... | | 1896 |
| American Ophthalmological Society, Transactions of the | Vol. VII., Part II. | 1896 |
| American Pediatric Society, Transactions of the ... ... | Vol. VII. | 1895 |
| American Surgical Association, Transactions of the... ... | Vol. XIII. | 1895 |
| Archivio di Ortopedia ... ... ... ... ... ... ... ... ... ... ... ... | | 1895 |
| Bristol and Clifton Directory ... ... ... ... ... ... ... ... ... ... | (7) | 1896 |
| Bristol Health Report ... ... ... ... ... ... ... ... ... ... ... ... | (2) | 1887 |
| Bristol Medico-Chirurgical Journal, The ... ... ... ... | Vol. XIII. | 1895 |
| Bristol Port Sanitary District—Annual Report of the Medical Officers of Health ... ... ... ... ... ... ... ... ... ... ... | (2) 1893; | 1896 |
| Bulletin de l'Académie de Médicine | 3e Sér. Tomes XXXIII., XXXIV. | 1895 |
| Bulletin de l'Académie royale de Médicine de Belgique ... | Tome IX. | 1895 |
| Canadian Practitioner, The ... ... ... ... ... ... ... ... ... | Vol. XX. | 1895 |
| Clinical Journal, The ... ... ... ... ... ... ... ... | Vol. VII. | 1896 |
| College of Physicians of Philadelphia, Transactions of the | 3rd Ser. Vol. XVII. | 1895 |
| Epidemiological Society of London, Transactions of the | N.S. Vol. XIV. | 1895 |
| Giornale Internazionale delle Scienze Mediche ... ... ... ... ... ... | | 1895 |
| Hospital, The ... ... ... ... ... ... ... ... ... ... ... | Vol. XIX. | 1896 |
| Hospitals— | | |
| Johns Hopkins Hospital Reports, The ... ... ... ... | Vol. IV. | 1895 |
| King's College Hospital Reports ... ... ... ... ... | Vol. II. | 1896 |
| Saint Bartholomew's Hospital Reports ... ... ... | Vol. XXXI. | 1895 |
| Saint Thomas's Hospital Reports ... ... ... | N.S. Vol. XXIII. | 1896 |
| Intercolonial Quarterly Journal of Medicine and Surgery ... | Vol. II. | 1896 |
| International Medical Magazine ... ... ... ... ... ... ... ... | Vol. IV. | 1896 |
| Johns Hopkins Hospital Bulletin, The... ... ... ... ... ... | Vol. VI. | 1895 |
| Laryngological Society of London, Proceedings of the | (3) Vols. I., II. | 1895-96 |
| Medical Annual, The ... ... ... ... ... ... ... ... ... ... ... ... | | 1896 |
| Medical News, The ... ... ... ... ... ... ... ... ... ... | Vol. LXVII. | 1895 |
| Medical Society of Virginia, Transactions of the ... ... ... ... ... | | 1895 |
| New Zealand Medical Journal, The ... ... ... ... ... ... | Vol. VIII. | 1895 |

| | | |
|---|---|---|
| Obstetrical Transactions ... ... ... ... ... ... ... | Vol. XXXVII. | 1896 |
| Royal Academy of Medicine in Ireland, Transactions of the (4) | Vol. XIII. | 1895 |
| Sei-I-Kwai Medical Journal, The ... ... ... ... ... ... | Vol. XIV. | 1895 |
| Union médicale, L' ... ... ... ... ... ... ... ... ... | 4e Sér. Tome I. | 1895 |
| Universal Medical Journal, The ... ... ... | N.S. Vols. I., 1893; III. | 1895 |
| Year-Book of Scientific and Learned Societies ... ... ... ... ... ... | | 1896 |

### Local Medical Notes.

### BATH.

W. H. Symons, M.D. Brux., D.P.H. Oxon, has been appointed Medical Officer of Health for the city, in place of Dr. Brabazon, deceased.

Mineral Water Hospital.—Preston King, B.A., M.D. Cantab, has been appointed Physician in place of Dr. Brabazon, deceased.

### BRISTOL.

Port Sanitary District.—Although there was no cholera scare during 1895, the medical officers of the port sanitary districts of the kingdom were on the alert to prevent the introduction of that disease. Now that quarantine has been abolished as unsatisfactory, constant and unremitting care and attention are necessary to carry out effectually the system now employed, for unless this work is done thoroughly it would be of no effect. The report of the Bristol Port Sanitary Authority has been distributed, and it shows how night and day ships coming to the port sanitary area of Bristol are being overhauled to discover the possible presence of infectious diseases on board. In addition to the ordinary infectious diseases, special attention was drawn during the year to the possible introduction of yellow fever from the port of Santos in Brazil, and though no one came under observation, disinfecting precautions were taken in one case. The report states that 1390 ships were inspected, of which 1106 were returned as good and 284 as unsatisfactory, showing on the whole improved sanitary conditions on board both foreign and coastwise vessels. Of the total number 781 were specially examined for cholera as they sailed from suspected ports, and in 37 cases it was found necessary to carry out the usual precautionary disinfecting measures. Only two ships arrived on which cases of cholera had actually occurred. Shipowners appear to fall in fairly readily with the required sanitary improvements suggested by the port authorities; but we notice that a considerable number of visits had to be paid before the requirements were carried out, and whether they are in all cases carried out cannot be stated, for the vessels have not all returned to the port of Bristol. Certain ships coming within the port sanitary area are bound for Gloucester, and by an arrangement with the authorities the cost of inspection is charged to that city. The steam launch at the disposal of the medical officers came to grief in September, and it was found impossible to perform the necessary work in the Severn in a row boat. This we can well believe. Only seven cases were admitted into the port hospital at Avonmouth during the year, of which all recovered.

The Degree of M.D. Durham has been conferred on C. E. Matthews, M.R.C.S. Eng., and on A. E. Blacker, L.R.C.S. Ed., and that of M.D. St. Andrews on Charles A. Hayman, L.R.C.S.I. At a quarterly meeting of the Comitia of the Royal College of Physicians of London on April 30th, John Michell Clarke, M.A., M.D. Cantab., Professor of Pathology at University College, was elected a Fellow.

University College.—C. A. La Touche Brough, LL.B., M.B., B.S. Durh., has been appointed Medical Tutor in place of A. E. H. Pinch, resigned. The following students have passed examinations: examining board in england by the royal colleges of physicians and surgeons: *First Examination. Part I. Chemistry and Physics*—H. G. Clark, S. L. Compton; *Part II. Practical Pharmacy*—E. Symes; *Part III. Elementary Biology*—J. A. Bartlett, L. A. Moore, J. C. Norton, G. S. Oades, A. M. S. Tordiffe, J. C. Wadmore, C. F. Walters. *Second Examination. Anatomy and Physiology*—E. G. Bunbury, J. R. Frost, H. M. Thomas. *Final Examination.* C. F. Druitt.* society of apothecaries: *Surgery*—A. F. Morgan.*

General Hospital.—Martin Randall, B.A., M.D. Lond., F.R.C.S. Eng., has been appointed House Surgeon.

Hospital for Sick Children and Women.—John Ewens, L.R.C.S., L.R.C.P. Edin., and T. Chalmers Norton, M.R.C.S. Eng., have resigned their posts as Surgeons and have been elected on the Consulting Staff. C. A. Morton, F.R.C.S. Eng., and H. W. Kendall, M.R.C.S. Eng., have been elected Surgeons. Colston Wintle, M.R.C.S. Eng., and Emily E. Eberle, M.A., M.B., B.Ch. R.U.I., have been elected Out-Patient Surgeons.

SWANSEA.

General and Eye Hospital.—R. C. Elsworth, M.B., C.M. Edin., M.R.C.S. Eng., has been appointed Surgeon, *vice* G. Herbert Hopkins, F.R.C.S., resigned. A. F. Blagdon Richards, M.A., M.B., B.C. Cantab., M.R.C.S., L.R.C.P. Lond., has been appointed Medical Officer to the Throat and Ear Department, *vice* R. C. Elsworth, resigned.

Institution for the Blind.—E. Le Cronier Lancaster, B.A., M.B., B.Ch. Oxon, M.R.C.S. Eng., has been appointed Hon. Physician, *vice* G. Herbert Hopkins, F.R.C.S., resigned.

* Completes Examination.

### PUBLICATIONS RECEIVED.

---

From Baillière, Tindall and Cox, London:
*Diseases of the Rectum.* By William Allingham and Herbert W. Allingham. Sixth Edition. 1896.
*The History of Surgical Anæsthesia.* By H. Bellamy Gardner. [1896.]

From John Bale & Sons, London:
*The Medical Inspection of, and Physical Education in, Schools.* By Charles Roberts. [1896.]

From Friedr. Bayer & Co., Elberfeld:
*Thyroïodin.* [n.d.]
*Latest Observations as to the Effect of Trional.* [1895.]

From Bennett Brothers, Ld., Bristol:
*Bristol Port Sanitary District. Annual Report of the Medical Officers of Health and of the Chief Port Inspector of Nuisances for the Year* 1895. 1896.

From T. Burleigh, London:
*State Aided* v. *Voluntary Hospitals.* By W. Knowsley Sibley, M.D. 1896.

From Cassell and Company, Limited, London:
*Surgical Diseases of the Ovaries and Fallopian Tubes, including Tubal Pregnancy.* By J. Bland Sutton. New and Enlarged Edition. 1896.

From The Church Defence Institution, London:
*The National Church.* April, 1896.

From J. & A. Churchill, London:
*Pharmacopœia of the Evelina Hospital for Sick Children.* Second Edition. 1896.
*Sterility.* By Robert Bell, M.D. 1896.
*The Contracted Granular Kidney.* By Sir George Johnson, M.D., F.R.S. 1896.

From Church Missionary Society, London:
*Medical Mission Quarterly.* No. XIV. April, 1896.

From The F. A. Davis Company, Philadelphia:
*Color-Vision and Color-Blindness.* By J. Ellis Jennings, M.D. 1896.
*Syphilis in the Middle Ages and in Modern Times.* By Dr. F. Buret. Translated by A. H. Ohmann-Dumesnil, M.D. 1895.
*Diseases of the Rectum and Anus.* By S. G. Gant, M.D. 1896.

From O. H. Dreyer, St. Louis:
*Medical Review.* March 14, 28; April 4, 11, 18; May 9, 16, 23, 30, 1896.

From Feret et Fils, Bordeaux:
*Du Traitement chirurgical de la Surdité et des Bourdonnements.* Dr. E. J. Moure. 1896.

From 1422 Folsom Street, San Francisco:
*California Medical Journal.* April, May, 1896.

From Dr. L. Webster Fox, Philadelphia:
*Evisceration of the Eyeball.* By L. Webster Fox, M.D. Third Paper. [n.d.] [Reprint.]
*Sarcoma of the Choroid, Glioma of the Retina, and New Formed Blood Vessels in the Vitreous.* By L. Webster Fox, M.D. [n.d.] [Reprint.]

From Headley Bros., London:
*On the Relation between Intemperance and Mental Disease.* By Bedford Pierce, M.D. 1895.

From M. Henry, London:
*Food and Sanitation.* March 14, 1896.

From W. H. Holloway, Newark, N.J.:
*Annual Report of the Directors and Medical Board of St. Michael's Hospital, under the Charge of the Sisters of the Poor of St. Francis, Newark, N.J.* 1896.

From G. Ibershoff, Oeynhausen:
*Oeynhausen und seine Indicationen.* [n.d.]

### PUBLICATIONS RECEIVED *(continued).*

From Iliffe & Son, Coventry:
*Our M.P.'s on the Cycle Tax.* 1896. [Reprint.]

From "Illustrated London News" Office, London:
*The English Illustrated Magazine.* April—June, 1896.

From Jeyes' Sanitary Compounds Co., Ltd., London:
*Lactophenine* (Hoechst). March, 1896.
*The Employment of Eudoxine in Intestinal Affections.* By Dr. T. Rosenheim. May, 1896.

From H. K. Lewis, London:
*Researches into the Anat my and Pathology of the Eye.* By E. Treacher Collins. 1896.
*Current from the Main.* By W. S. Hedley, M.D. 1896.
*Public Health Laboratory Work.* By Henry R. Kenwood, M.B. Second Edition. 1896.
*Rough Notes on Remedies.* By Wm. Murray, M.D. 1896.
*Hydro-Electric Methods.* By W. S. Hedley, M.D. Second Edition. 1896.
*Lewis's Diet Charts.*

From James Maclehose and Sons, Glasgow:
*Memorials of the Faculty of Physicians and Surgeons, Glasgow.* By Alexander Duncan, B.A. 1896.

From G. Mellin, London:
*The Feeding of Infants and Growing Children.* Edited by G. Mellin. 1895.

From W. J. P. Monckton, London:
*The Ludgate.* No. 7, Vol. II. May, 1896.

From Oliver and Boyd, Edinburgh:
*Curettage of the Uterus: History, Indications, and Technique.* By J. W. Ballantyne, M.D. 1896.
*Congenital Teeth, with Three Illustrative Cases.* By J. W. Ballantyne, M.D. 1896. [Reprint.]

From The Rebman Publishing Company, Limited, London:
*An American Text-Book of Obstetrics.* Richard C. Norris, M.D., Editor. In two parts. 1896.
*Archives of Clinical Skiagraphy.* By Sydney Rowland, B.A. Vol. I., Part 1. May, 1896.

From John Morgan Richards, London:
*Medical Reprints.* March—May, 1896.

From Dr. Hunter Robb, Cleveland:
*Pruritus of the Genitals.* By Hunter Robb, M.D. 1896. [Reprint.]

From The Roxburghe Press, London:
*The Nursing News and Hospital Review.* March, 1896.

From W. B. Saunders, Philadelphia:
*Diets for Children.* By Louis Starr, M.D. 1896.
*Obstetric Accidents, Emergencies, and Operations.* By L. Ch. Boisliniere, A.M., M.D., LL.D. 1896.

From The Scientific Press, Limited, London:
*Elementary Anatomy and Surgery for Nurses.* By W. McAdam Eccles, M.S. 1896.
*Clinical Diagnosis.* By W. G. Aitchison Robertson, M.D., D.Sc. 1896.

From 1 Southwark Street, London:
*The First Annual Report of the Clinical Research Association, Limited.* 1896.

From Det Steenske Bogtrykkeri, Christiania:
*Kastration ved Prostatahypertrofi.* Af Fredrik Ramm. 1896.

From Elliot Stock, London:
*Dress and Health.* By Charles Moore Jessop. 1896.

From George Tiemann & Co., New York:

*Tiemann's Reprints.* No. 15. [N.D.]

From The United Kingdom Alliance, Manchester:
*Doctors and Drinking.* Compiled by R. A. Jameson. 1896.

From Dr. F. H. Wiggin, New York:
*The Necessity of Complete Extirpation of Tumors and the Importance of Rapid Cicatrization of the Wound.* By Frederick Holme Wiggin, M.D. 1895. [Reprint.]

From John Wright & Co., Bristol:
*A Text-book of Histology.* By Arthur Clarkson, M.B., C.M. 1896.

From J. Zaehnsdorf, London:

*A Short History of Bookbinding.* 1895.

